# Recent insights into the biological functions of apigenin

**DOI:** 10.17179/excli2020-2579

**Published:** 2020-07-06

**Authors:** Jae Kwang Kim, Sang Un Park

**Affiliations:** 1 Division of Life Sciences and Bio-Resource and Environmental Center, Incheon National University, Incheon 22012, Korea; 2 Department of Crop Science, Chungnam National University, 99 Daehak-ro, Yuseong-gu, Daejeon, 34134, Korea

## ⁯

***Dear Editor,***

Apigenin (4′,5,7-trihydroxyflavone) belongs to the group of flavonoids positioned on the backbone of 2-phenylchromen-4-one (2-phenyl-1-benzopyran-4-one) and is most extensively allocated in herbs, vegetables, and fruits (Shankar et al., 2017[46]; Sharma et al., 2019[47]). Biosynthetically, apigenin is obtained from the phenylpropanoid pathway and also from the flavone synthesis pathway (Forkmann, 1991[12]) The pathway of phenylpropanoid begins from the aromatic amino acids L-phenylalanine or L-tyrosine, both products of the shikimate pathway (Herrmann, 1995[18]).

In several recent studies, it has been shown that apigenin has a number of valuable bioactive functions, including antibacterial, antiviral, antiproliferative, anti-inflammatory, antioxidant, antiangiogenic, and anticancer activities (Kowalczyk et al., 2017[25]; Nabavi et al., 2018[35]; Ghițu et al., 2019[14]).

From the results of several *in vivo* and* in vitro* studies and clinical trials, apigenin has been shown to be an effective curative treatment for rheumatoid arthritis, autoimmune disorders, Parkinson's disease, Alzheimer's disease, and several types of cancers (Tang et al., 2017[51]; Salehi et al., 2019[43]). Here, we summarize the key findings of the biological and pharmacological actions of apigenin (Table 1(Tab. 1); References in Table 1: Ahmad et al., 2019[1]; Ai et al., 2017[2]; Amiri et al., 2018[3]; Britto et al., 2017[4]; Charalabopoulos et al., 2019[5]; Chen et al., 2017[7], 2019[8], 2020[6]; Choi et al., 2018[9]; Dean et al., 2018[10]; Feng et al., 2017[11]; Ganai, 2017[13]; Han et al., 2017[15]; Hassan et al., 2017[16]; He et al., 2020[17]; Huang et al., 2020[19]; Jiang et al., 2018[20]; Jiao et al., 2019[21]; Jing et al., 2019[22]; Kang et al., 2018[23]; Ketkaew et al., 2017[24]; Lee et al., 2019[26]; Li et al., 2017[28], 2019[29], 2020[27]; Liu et al., 2018[30]; Lu et al., 2019[31]; Malik et al., 2017[32]; Mirzoeva et al., 2018[33]; Mrazek et al., 2019[34]; Nelson et al., 2017[36]; Pang et al., 2019[37]; Qiu et al., 2019[38]; Quan et al., 2020[39]; Rašković et al., 2017[40]; Ren et al., 2018[41]; Safari et al., 2018[42]; Sánchez-Marzo et al., 2019[44]; Sang et al., 2017[45]; Sharma et al., 2018[48]; Siddique and Jyoti, 2017[49]; Stump et al., 2017[50]; Thangaiyan et al., 2018[52]; Tong et al., 2019[53]; Wang et al., 2017[54], 2018[56], 2019[55]; Wu et al., 2017[57]; Xu et al., 2018[58]; Zare et al., 2019[59]; Zhang et al., 2017[61], 2018[63], 2019[62], 2020[60]; Zhao et al., 2019[64]; Zhong et al., 2017[65], 2018[66]; Zhou et al., 2019[67]).

| Key findings | Reference |
| --- | --- |
| Apigenin retards the growth of tumor during the estrogen receptor-mediated PI3K/Akt/mTOR pathway and also acts to reduce the effects of histamine on tumors. | Zhang et al., 2020 |
| The alteration of both apigenin and luteolin signaling pathways acts to inhibit IL-31 and IL-33, which indicates the importance of these two cytokines in neurodegenerative diseases. | Chen et al., 2020 |
| Apigenin enhances the anti-lymphoma effect of Bruton's tyrosine kinase inhibitors and imparts to targeted therapy of those arise resistance. | Huang et al., 2020 |
| It has been proposed that apigenin could act to reduce pulmonary hypertension by inhibiting the hypoxia-induced factor-1α-KV1.5 channel pathway to provoke pulmonary artery smooth muscle cell (PASMC) mitochondria-dependent apoptosis. | He et al., 2020 |
| Apigenin has been recognized as a factor in the treatment of ischemic heart disease, as it has a protective effect against myocardial ischemia/reperfusion injury in rats and oxygen and glucose deprived/reoxygenation-induced injury in cardiomyocytes by suppressing translocation of NF-κB, thus reducing the inflammatory response. | Quan et al., 2020 |
| Apigenin has been shown to be effective in repressing the formation of abdominal aortic aneurysms (AAA) by inhibiting the NF-κB signaling pathway, which suggests that apigenin might have potential as a therapeutic drug in prevention of AAA. | Li et al., 2020 |
| It is noted that apigenin could enhance lipid metabolism by activating the AMP-activated protein kinase-sterol regulatory element-binding protein (AMPK-SREBP) pathway to reduce lipid accumulation in the liver. | Lu et al., 2019 |
| Both anti-allergic and anti-neutrophil-related inflammatory effects have been observed when apigenin is used as a treatment in a murine asthma model under particulate matter of PM2.5, perhaps by changing IL-17 and down-regulating the NF-κB expression. Therefore, apigenin can be seen as a candidate to treat PM2.5 exposure-enhanced pre-existing asthma. | Pang et al., 2019 |
| Apigenin is recognized as a novel IL-6 transcription inhibitor with possible anti-cancer effects. The application of apigenin in the clinical treatment of esophageal cancer warrants further study. | Qiu et al., 2019 |
| Apigenin can slow the proliferation of fibroblast and decrease epidural fibrosis by repressing the Wnt3a/β-catenin signaling pathway. This may be a new approach to prevent or delay the onset of epidural fibrosis. | Jiao et al., 2019 |
| Apigenin could help in cases of ACN (acrylonitrile)-induced neurotoxicity in the brain by inhibiting the TLR4/NF (necrosis factor)-κB signaling pathway and could demonstrate additional neuroprotective effects. | Zhao et al., 2019 |
| Through the AMPK/mTOR (mammalian target of rapamycin) pathway, apigenin actively supports autophagy, thus providing anti-depressive effects in chronic restraint stressed mice. | Zhang et al., 2019 |
| Apigenin has an anti-invasive effect in the breast cancer cell line MDA-MB-231-derived xenograft tumors, by hindering the IL-6-linked downstream signaling pathway. | Lee et al., 2019 |
| The application of apigenin resulted in reduced growth of cisplatin-resistant colon cancer cells both *in vitro* and *in vivo.* Apigenin might be utilized to enhance the therapy of colon cancer. | Chen et al., 2019 |
| Apigenin reduces contractions induced by several vasoconstrictors in rat intrarenal arteries (RIRA) by suppressing CaCC ($Ca^{2+}$ activated $Cl^-$ channels) currents and augmenting Kv (voltage-dependent) currents in RIRA arterial smooth muscle cells. | Jing et al., 2019 |
| From the clinical trial both in *in vitro* and *in vivo*, it has been reported that apigenin prevented the epithelial-mesenchymal transition, migration, and invasion of human colon cancer cells through the NF-κB/Snail pathway. | Tong et al., 2019 |
| Apigenin can slow the progression and lessen the severity of acute pancreatitis. Apigenin can also serve as a supplemental therapeutic strategy in critical pancreatitis. | Charalabopoulos et al., 2019 |

EXCLI Journal 2020;19:984-991 – ISSN 1611-2156
Received: June 27, 2020, accepted: July 02, 2020, published: July 06, 2020

| **Key findings** | **Reference** |
|---|---|
| Recent studies have shown that apigenin can easily decrease cardiac injuries provoked by doxorubicin through anti-fibrotic, antioxidant, and anti-apoptotic properties. It appears that apigenin inhibits cardiac injuries and helps to recover cardiac function. | Zare et al., 2019 |
| Very recently, it was reported that apigenin apparently slows the growth and migration of hypertrophic scar fibroblasts by reducing focal adhesion kinase signaling. From this finding, it can be proposed that apigenin might be a new option for the treatment of hypertrophic scars. | Wang et al., 2019 |
| Apigenin can be used as a potential agent to improve health disorders resulted from advanced glycation end products by changing cellular inflammatory and antioxidant defense signaling pathways. | Zhou et al., 2019 |
| Apigenin protects against collagen-induced arthritis by suppressing synovial hyperplasia, angiogenesis, and osteoclastogenesis. Apigenin can be considered as a putative low-toxicity candidate drug for rheumatoid arthritis treatment. | Li et al., 2019 |
| It is reported that apigenin lowered inflammatory responses in experimental models of recurrent acute pancreatitis. The mechanisms intervene the actions of apigenin, in part, are outstanding to decrease parathyroid hormone-related protein and transforming growth factor β pro inflammatory signaling. | Mrazek et al., 2019 |
| Due to the ability to protect against UVA and UVB-induced skin damage, apigenins could be interesting candidates for the improvement of oral and topical photoprotective ingredients. In particular, the capability of apigenin-K to increase water solubility makes it the best candidate for further development. | Sánchez-Marzo et al., 2019 |
| Apigenin could be a strong dietary anti-oxidant regimen against EDF-induced toxicity through its activities against cytotoxicity and DNA damage in lymphocytes. | Ahmad et al., 2019 |
| Apigenin showed a positive response against paraquat-induced acute lung injury as well as immunotoxicity by modulating inflammation and oxidative stress. | Liu et al., 2018 |
| Melanoma growth could be restricted by treatment with apigenin, which exhibits multiple mechanisms through repression of programmed cell death ligand-1 expression. | Xu et al., 2018 |
| Both *in vivo* and *in vitro* trials of apigenin revealed that it can defend renal function against ischemia/reperfusion injury and also inhibit the growth of renal tubular cells from apoptosis through phosphoinositide 3-kinase (PI3K)/Akt mediated mitochondria-dependent apoptosis signaling pathway. | Wang et al., 2018 |
| It is reported that apigenin could protect against isoproterenol hydrochloride-induced antioxidant depletion, oxidative DNA damage, and inflammatory and apoptotic signaling in H9C2 cells. These findings suggest that apigenin has a cardioprotective effect on cardiomyoblast cells. | Thangaiyan et al., 2018 |
| The cutaneous tumor suppressor thrombospondin-1 is considered a key mediator of the anticancer effect of apigenin *in vivo* on skin, particularly showing its anti-inflammatory property. | Mirzoeva et al., 2018 |
| Apigenin exerts a strong protective effect against 3-chloro-1, 2-propanediol (3-MCPD)-induced renal injury by modifying the mitochondria-dependent caspase cascade pathway. | Zhong et al., 2018 |
| It is concluded that apigenin impaired atherogenesis by up-regulating ATP binding cassette A1-mediated cholesterol efflux, thus hindering inflammation. | Ren et al., 2018 |
| Treating with apigenin can successfully control mouse mesothelial peritoneal cell fibrosis induced by high glucose, regulated by the suppression of miR34a expression. | Zhang et al., 2018 |
| Recently it was reported that apigenin was able to protect mouse embryos against $H_2O_2$ and actinomycin D, as evidenced by the increase in the number of viable blastomeres and decrease in the number of apoptotic blastomeres. The mechanisms of increasing the rate of hatching in mouse embryos through expanding the blastocysts, thinning of the zona pellucida thickness. | Safari et al., 2018 |
| Through the activation of the Nrf2 (nuclear factor erythroid-derived 2-related factor 2) signaling pathway and inhibition of the NF-κB signaling pathway, apigenin can protect against lipopolysaccharide-induced endometritis. | Jiang et al., 2018 |

| **Key findings** | **Reference** |
|---|---|
| Treatment with apigenin might reduce cognitive deficits and reverse behavior impairments, without changing seizure acuteness in kindled mice. These effects can be attributed to cAMP response element binding protein / brain-derived neurotrophic factor upregulation in the hippocampus. | Sharma et al., 2018 |
| *In vivo* trials of apigenin showed efficient anti-plasmodial action against *Plasmodium berghei* infection in mice, with high selectivity against malaria. | Amiri et al., 2018 |
| Apigenin can act as a phytoprogestin with mixed agonist activity *in vivo* by impeding estrogen receptor-mediated uterine proliferation. | Dean et al., 2018 |
| Apigenin can apparently act to inhibit denervation-induced muscle atrophy, through the inhibitory effect on inflammatory processes within muscle. | Choi et al., 2018 |
| Apigenin can be used to improve death receptor 5 apoptosis by stimulating apoptotic caspases through upregulating death receptor 5 expression, regardless of ROS generation. This may be  a promising strategy for an adjuvant of TRAIL. | Kang et al., 2018 |
| Through suppressing STAT3-NF-κB signaling, apigenin is considered to inhibit inflammation and inflammation-induced carcinogenesis in inflammatory bowel disease and colitis-associated cancer. | Ai et al., 2017 |
| Apigenin can be used as a potent bioactive agent to increase dopamine content, reduce oxidative stress as well as apoptosis, and prolong life in transgenic *Drosophila* model of Parkinson's disease. | Siddique and Jyoti, 2017 |
| It is noted that apigenin showed a defensive response to alcohol-induced liver injury, which could be explained by the regulation of hepatic cytochrome P450 2E1-mediated oxidative stress and peroxisome proliferator-activated receptor alpha-mediated lipogenic gene expression. | Wang et al., 2017 |
| It has been determined that memory impairment induced by isoflurane exposure is closely related to dysregulated histone acetylation in the hippocampus, which influences brain-derived neurotrophic (BDNF) expression and hence the BDNF downstream signaling pathway. Apigenin improves cognitive function by restoring histone acetylation and suppressing neuroinflammation. | Chen et al., 2017 |
| Apigenin has been shown to defend skin cells through its sunscreen effect against UVB-induced cyclobutane pyrimidine dimers formation. For this reason, apigenin can be designated as an efficient means of protection against UV-induced skin damage. | Britto et al., 2017 |
| By amending ROS-mediated mitochondrial dysfunction and the caspase signal pathway, apigenin protects against patulin-induced apoptosis in HEK293 cells. | Zhong et al., 2017 |
| Apigenin may show anti-senescent activity by activating the Nrf2 pathway. Such a mechanism could be an effective strategy for delaying the aging process. | Sang et al., 2017 |
| Apigenin can improve renal damage due to DN (diabetic nephropathy) by suppressing oxidative stress and fibrosis and also by slowing the MAPK pathway. | Malik et al., 2017 |
| Apigenin can contribute to the reduction in blood fat *in vivo* through enhancing the absorption and conversion of cholesterol and hastening reverse cholesterol transport. It is also reported that apigenin has a role in fighting oxidization and protecting blood vessels. | Zhang et al., 2017 |
| Apigenin can reverse nonalcoholic fatty liver disease through a new regulating mode of Nrf2 and peroxisome proliferator-activated receptor gamma by reducing lipid metabolism and oxidative stress abnormity. | Feng et al., 2017 |
| Apigenin might have potential as a new drug for the treatment of hepatocellular carcinoma. The technology of optical molecular imaging permitted the non-invasive and reliable assessment of anti-tumor drug efficacy in liver cancer. | Li et al., 2017 |
| Apigenin exhibited a potent inhibitory effect on pathways involved in glioblastoma (GBM) proliferation and survival and might effectively be used as a therapeutic agent for GBM. | Stump et al., 2017 |
| Apigenin could have a neuroprotective effect, as it shows both anti-oxidation and anti-apoptosis activity. These findings form a theoretical foundation on which an effective drug for treatment of subarachnoid hemorrhage can be based. | Han et al., 2017 |

| Key findings | Reference |
|---|---|
| It is reported that apigenin can control the level of lipid peroxidation and considerably increase the enzyme antioxidant mechanisms in paracetamol-induced hepatotoxicity in rats. | Rašković et al., 2017 |
| To maintain T cell homeostasis in murine pancreatic cancer, apigenin may be a promising therapeutic agent, by stabilizing Ikaros expression and function. | Nelson et al., 2017 |
| Due to the antioxidant and anti-inflammatory effects of apigenin, it can be considered a protective and promising preventive strategy against cisplatin-induced nephrotoxicity. | Hassan et al., 2017 |
| By reducing the promoter activities of two viral IE (immediate-early) genes, apigenin protects against Epstein-Barr virus (EBV) reactivation and can be considered a potential dietary agent for prevention of EBV reactivation. | Wu et al., 2017 |
| Compared with conventional therapies, apigenin demonstrates synergistic effects in combinatorial therapy and stimulates apoptosis in prostate cancer models. | Ganai, 2017 |
| Apigenin inhibits the expression of cancer stem cell markers and also the number of cells expressing cell surface markers under hypoxia. | Ketkaew et al., 2017 |

## *Conflict of interest*

The authors declare no conflict of interest.

## Acknowledgements

This research was supported by Golden Seed Project (213006051WTE11) funded by the Ministry of Agriculture, Food and Rural Affairs (MAFRA), the Ministry of Oceans and Fisheries (MOF), the Rural Development Administration (RDA), and the Korea Forest Service (KFS), Republic of Korea.

## Conflict of interest

The authors declare no conflict of interest.

## Figures and Tables

**Table 1:**
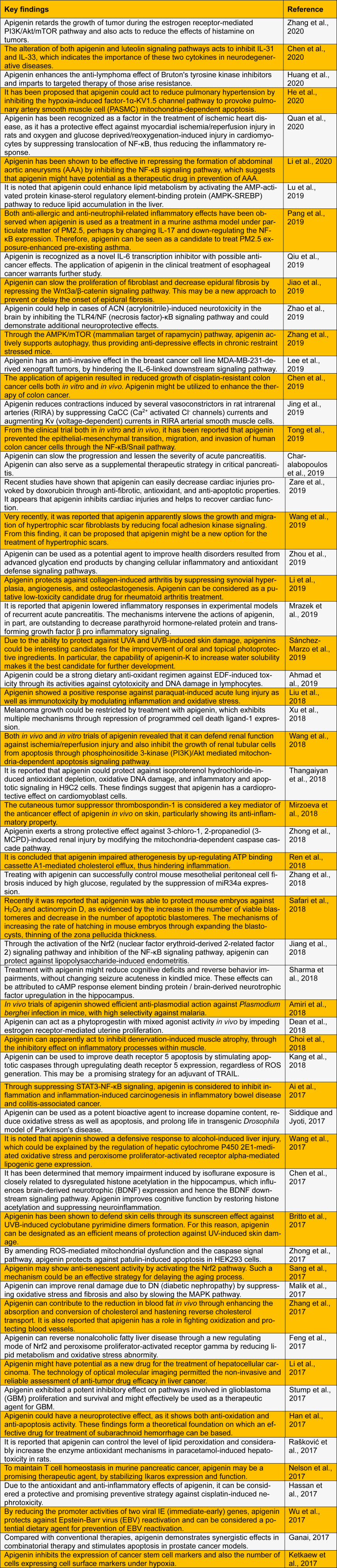
Recent studies of the biological and pharmacological activities of apigenin
